# Hollow Gold–Silver Nanorods—A New, Very Efficient Nanomaterial for Surface-Enhanced Raman Scattering (SERS) Measurements

**DOI:** 10.3390/molecules29194540

**Published:** 2024-09-25

**Authors:** Aleksandra Michałowska, Andrzej Kudelski

**Affiliations:** Faculty of Chemistry, University of Warsaw, Pasteura 1 Str., PL 02-093 Warsaw, Poland; a.michalowska10@uw.edu.pl

**Keywords:** hollow plasmonic nanoparticles, SERS spectroscopy, nanoresonators

## Abstract

Anisotropic plasmonic nanoparticles usually generate SERS enhancement factors that are significantly larger than those generated by spherical plasmonic nanostructures, so the former are usually preferred as substrates for SERS measurements. Gold nanorods are one of the most commonly used anisotropic nanomaterials for SERS experiments. Unfortunately, even a slight contamination of the surfactant used in the process of the synthesis of gold nanorods has a significant impact on the geometry of the resulting nanostructures. In this work, using easily formed silver nanorods as templates, hollow AuAg nanorods are formed by means of a silver–gold galvanic exchange reaction (in this process, nanostructures with a cavity inside form because one gold atom replaces three silver atoms). Hollow AuAg nanorods are highly active during SERS measurements—for shorter wavelengths of the excitation radiation, they display greater SERS activity than Au nanorods. To our knowledge, this is the first example of the use of hollow plasmonic nanorods for SERS measurements. Elemental mapping of the rods showed that the silver, some of which remained after the galvanic replacement, is mainly located close to the internal cavity that was formed, whereas the gold is mainly located at the outermost regions of the nanostructure. This explains the high chemical stability of these nanostructures.

## 1. Introduction

Standard Raman spectroscopy is not considered as an especially sensitive analytical tool because a typical total Raman scattering cross-section is only ca. 10^−29^ cm^2^ per molecule, whereas, for comparison, the typical cross-sections for the absorption of ultraviolet and infrared radiation are ca. 10^−18^ and 10^−21^ cm^2^ per molecule, respectively [1]. This means that in a standard Raman analysis, one usually has to use a solution of an analyte having a concentration larger than ca. 10^−3^ M (otherwise, no reliable spectra are obtained). This limitation of Raman spectroscopy can be overcome, however, by depositing the analytes on certain plasmonic nanostructures, or by placing the plasmonic nanostructures on the analyzed surfaces [1]. When illuminated, the plasmonic nanostructures generate a local enhancement of the intensity of the incident electric field, and also generate an enhancement of the intensity of the scattered radiation. This can lead to a very large increase in the efficiency of the generation of the Raman signal in an effect known as surface-enhanced Raman scattering (SERS) [1]. In some cases, the efficiency of the generation of the Raman signal via the SERS effect is so large that it is possible to observe a high-quality spectrum of even a single molecule [2,3,4]. This feature makes SERS one of the most sensitive analytical tools known. 

To generate large SERS enhancement factors, one has to use appropriate plasmonic nanostructures that significantly increase the local intensity of the electric field of the excitation radiation and also enhance the intensity of the scattered radiation (a description of exemplary substrates for SERS measurements can be found in Refs. [5,6,7]). Previous experiments and theoretical simulations have shown that anisotropic plasmonic nanoparticles usually generate larger SERS enhancement factors than spherical plasmonic structures [8,9,10]. Therefore, many types of anisotropic plasmonic nanoparticles have been used to construct SERS-active materials, for example, nanoparticles in the shape of cubes [11,12,13], triangular prisms [14,15], various bipyramids [16,17,18,19], stars [20,21,22], or rods [23,24,25,26]. Plasmonic nanorods seem to be a particularly promising nanomaterial for SERS measurements because their plasmon resonance can easily be tuned over a wide range of frequencies by changing their size and aspect ratio [27]. In the case of SERS measurements using systems containing many aggregated plasmonic nanostructures, very large enhancements of the intensity of the electric field, and hence, very large SERS enhancement factors, are generated in the narrow slits between the plasmonic objects [8,9,10]. However, in these cases as well, the SERS substrates formed from anisotropic plasmonic nanostructures are usually significantly more efficient in increasing the efficiency of the Raman signal generation than substrates formed from isotropic plasmonic nanoparticles.

**Figure 1:**
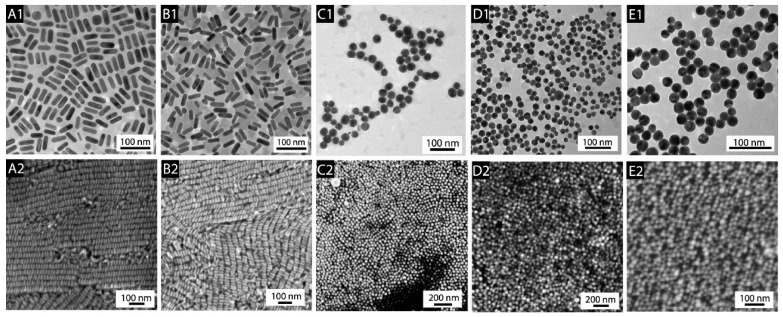
TEM (top images labelled with “1”) and SEM (bottom images labelled with “2”) images of gold nanostructures synthesized using CTAB (hexadecylcetyltrimethylammonium bromide) from five different suppliers (experiments carried out in 2007). (**A1**,**A2**) Fluka (product: 52370), (**B1**,**B2**) MP Biomedicals (product: 194004), (**C1**,**C2**) Acros (product: 22716V), (**D1**,**D2**) Sigma (product: H5882), and (**E1**,**E2**) Aldrich (product: 855820). Among the tested samples of CTAB, only the CTAB from two suppliers yielded nanorods, while the others yielded only spherical nanoparticles; for details, see Ref. [28]. Reprinted with permission from American Chemical Society from Ref. [28]. Copyright 2008, American Chemical Society.

One of the most commonly used anisotropic nanomaterials for SERS experiments is the gold nanorods. Unfortunately, even a slight contamination of the surfactant used in the process of the synthesis of gold nanorods has a significant impact on the geometry of the nanostructures that form. An example of how changing the supplier of the surfactant used in the synthesis affects the geometry of the resulting product is shown in Figure 1 [28]. On the other hand, in the case of the synthesis of silver nanorods, such a strong effect of the influence of surfactant impurities on the geometries of the resulting nanostructures is not observed, but silver nanorods, unfortunately, are much less chemically stable (which is particularly important when studying biological systems), and silver also has significant biotoxicity. In this work, we look at very promising and easily formed nanorods for SERS measurements: hollow AuAg nanorods. These can be easily formed from silver nanorods through a silver–gold galvanic exchange reaction. Moreover, elemental analysis of the resulting hollow AuAg nanorods showed that the silver, some of which remained after the galvanic replacement, is mainly located close to the internal cavity that was formed, whereas the outermost part of the nanostructure is composed mainly of gold. This makes such nanostructures suitable for biological SERS experiments.

## 2. Results and Discussion

### 2.1. Structural Characterization of the Obtained Nanomaterials

**Figure 2:**
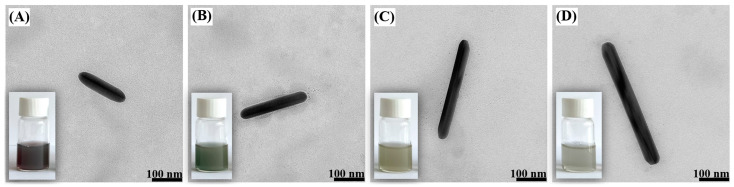
TEM images of Ag nanorods obtained using different amounts of seed solution: (**A**) 0.5 mL, (**B**) 0.25 mL, (**C**) 0.125 mL and (**D**) 0.06 mL. The insets at the bottom left are photos of the actual sols of silver nanorods.

TEM images of different Ag nanorods (used as templates for the formation of the AuAg nanorods) are shown in Figure 2. Their length can be changed by changing the ratio of the mixed seed and growth solutions—when a smaller quantity of the seed solution was added, there was a noticeable increase in the length of the Ag nanorods. The color of the sols of silver nanorods exhibited various shades of green, as shown in the lower left corner of the insets accompanying each TEM image. This color variation stems from differences in the morphology and composition of the nanorod, providing valuable visual cues as characteristics.

**Figure 3:**
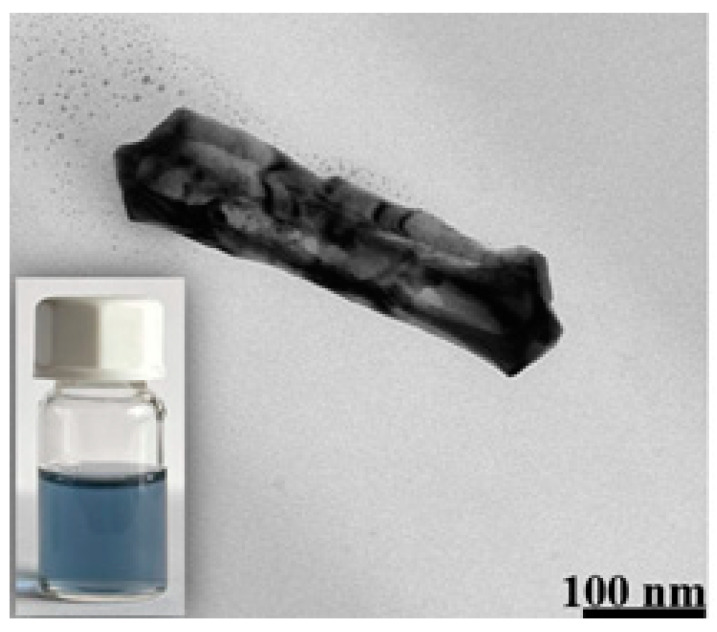
TEM image of a formed AuAg nanorod. The inset at the bottom left is a photo of the actual sol of the nanostructures.

**Figure 4:**
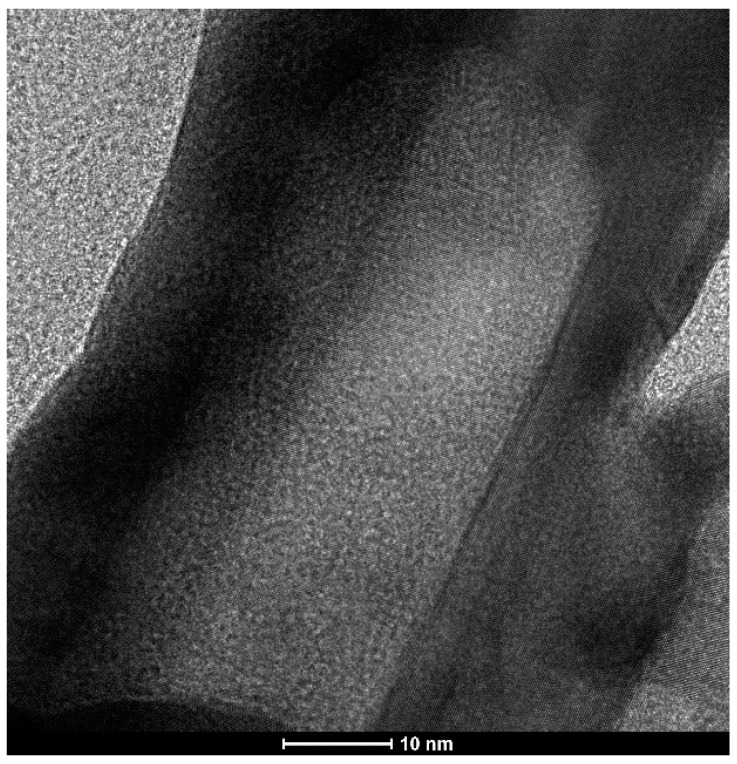
High-resolution TEM (HRTEM) image of a formed AuAg nanorod.

The addition of HAuCl_4_ to the sol of silver nanorods caused a silver–gold galvanic exchange reaction to occur. Because one gold atom replaces three silver atoms (as a result of the galvanic exchange reaction, a gold compound in the +3 oxidation state is reduced to metallic gold, while metallic silver is oxidized to a silver compound in the +1 oxidation state), hollow nanorods are formed; see Figure 3 and Figure 4 (the empty central region is visible as a brighter contrast region within the nanorod). More images of the AuAg nanostructures that were formed are shown in the paragraph presenting elemental composition maps of the nanostructures.

**Figure 5:**
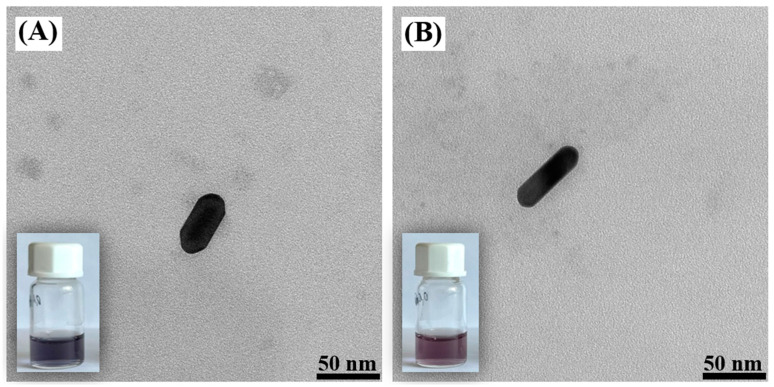
TEM images of Au nanorods. An Au nanorod with length of (**A**) 45 nm and (**B**) 65 nm The insets at the bottom left are photos of sols containing the gold nanorods.

In order to perform SERS measurements, Au nanorods with lengths of 45 and 65 nm were also synthesized. Sample TEM images of those nanoparticles are shown in Figure 5.

### 2.2. Elemental Characterization of AuAg Nanorods

**Figure 6:**
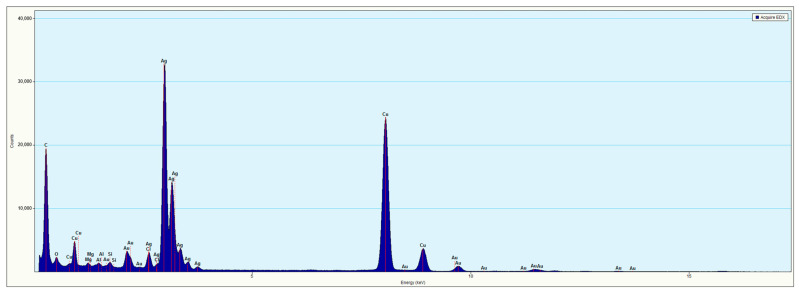
EDS spectrum of the AuAg nanorods that were obtained.

Energy-dispersive spectroscopy (EDS) measurements were made to analyze the elemental composition of the hollow AuAg nanorods that were formed (a sample EDS spectrum of the AuAg nanorods that were obtained is shown in Figure 6). 

**Figure 7:**
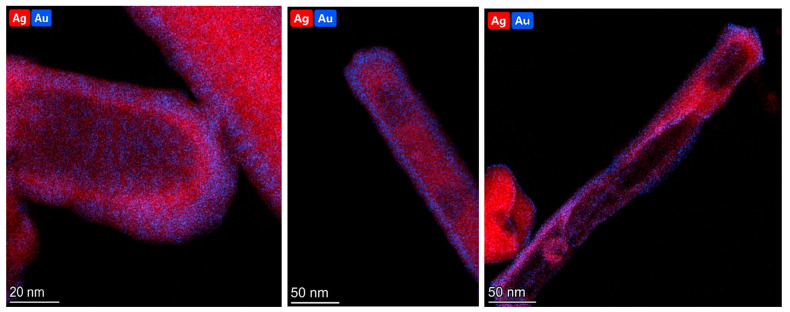
Sample EDS maps showing the topographic distribution of silver (red dots) and gold (blue dots) in the AuAg nanorods.

The EDS analysis confirmed the presence of both silver and gold within the synthesized nanostructures. However, the distribution of gold and silver was not uniform (see Figure 7). The gold content was definitely higher in the outermost parts of the nanostructures (which were the easiest for the chloroauric acid to reach). Silver was mainly located close to the wall of the internal cavity that was formed (see Figure 7).

### 2.3. Optical Characterization of the Nanorods

**Figure 8:**
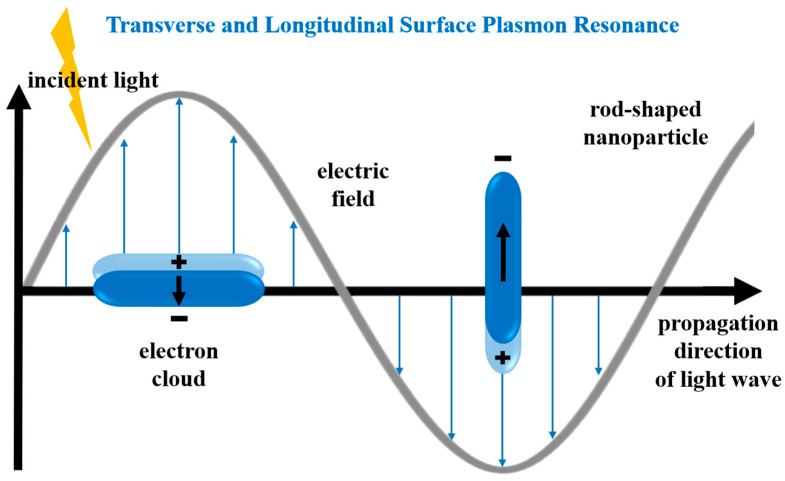
Schematic illustration of transverse and longitudinal surface plasmon resonance in a rod-shaped plasmonic nanoparticle.

An optical characterization of the rod-shaped nanostructures that were obtained was performed using UV–vis extinction spectroscopy. In the UV–vis extinction spectra of the sols of the solid plasmonic nanorods, one can observe two prominent bands: a longitudinal resonance band appearing at longer wavelengths, which is associated with the oscillation of free electrons along the long axis of the nanorods, and a transverse resonance band seen at shorter wavelengths, corresponding to the electron oscillations perpendicular to the long axis of the nanorods (see Figure 8).

**Figure 9:**
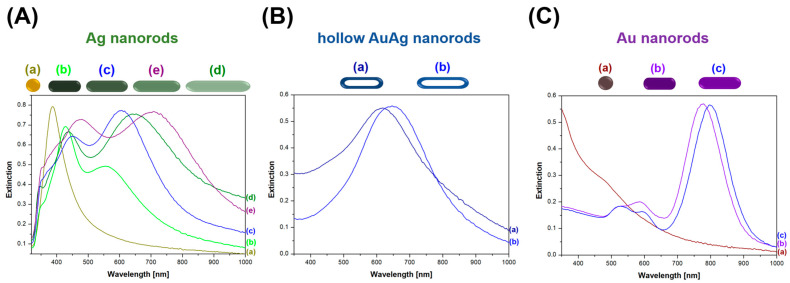
UV−vis extinction spectra of (**A**) various Ag nanorods, (**B**) various hollow AuAg nanorods and (**C**) various Au nanorods.

Both transverse and longitudinal plasmonic bands are visible in the extinction spectra of the sols of the solid silver and gold nanorods (see Figure 9A,C); because of the significantly larger aspect ratio in the synthesized solid Ag nanorods than in the solid Au nanorods, both bands (longitudinal and transverse) are more clearly visible in the extinction spectra of the sols of Ag nanorods (see Figure 9A). As can be seen in Figure 9A,C, as the aspect ratio of the solid plasmonic nanorods increases, the longitudinal resonance band clearly shifts towards longer wavelengths. The position of the transverse band changes to a much smaller extent than the position of the longitudinal band. Interestingly, for the hollow AuAg nanorods, no noticeable transverse band was observed (see Figure 9B). This effect is related to the fact that the interior of the hollow nanostructures is not conductive, so the vibrations of free electrons in the hollow nanorods must be realised in a slightly different way than in the solid nanostructures.

Hollow AuAg nanorods are formed using solid Ag nanorods as templates. We decided to check how the position of the longitudinal resonance plasmonic band for a specific sample of solid Ag nanorods shifts as a result of the galvanic exchange reaction. In all the experiments that were carried out, the galvanic AuAg exchange process led to a shift in the longitudinal plasmonic band towards longer wavelengths. For example, using Ag nanorods with a longitudinal plasmonic band at 563 nm as templates, the AuAg nanorods that were formed showed a maximum extinction at 617 nm. When Ag nanorods with a longitudinal plasmonic band positioned at 603 nm were used as templates, the AuAg nanorods showed a maximum extinction at 642 nm. 

### 2.4. SERS Measurements

The high aspect ratio of nanorods, which are characterized by an elongated shape and nanoscale dimensions, facilitates the generation of strong electromagnetic fields at their tips. Therefore, plasmonic nanorods are often used as substrates to carry out SERS measurements because they generate significantly larger SERS enhancement factors than standard spherical plasmonic nanoparticles. This helps increase the sensitivity of the SERS measurements.

**Figure 10:**
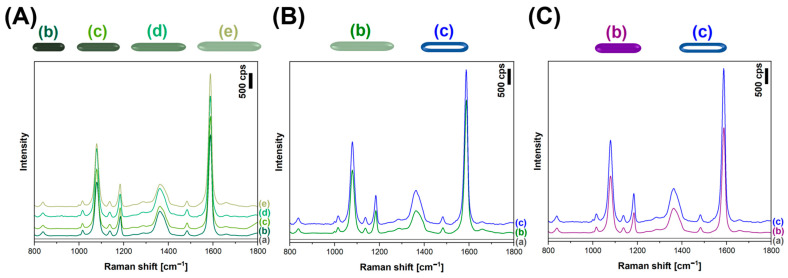
(**A**–**C**): (a) Raman spectra of a p-MBA monolayer on Pt before the deposition of plasmonic nanorods. (**A**): (b–e) Raman spectra of a p-MBA monolayer on Pt covered with various solid silver nanorods; (**B**) Raman spectra of a p-MBA monolayer on Pt covered with (b) solid Ag nanorods and (c) hollow AuAg nanorods; (**C**) Raman spectra of a p-MBA monolayer on Pt covered with (b) solid Au nanorods and (c) hollow AuAg nanorods. Excitation radiation wavelength: 633 nm.

In order to check the activity of the various types of Ag, Au and AuAg nanorods from this work using SERS measurements, we deposited samples of different nanorods that were similar (in terms of the number of moles of plasmonic metal used) onto a monolayer of 4-mercaptobenzoic acid (p-MBA) chemisorbed on the surface of platinum. As can be seen in Figure 10, when excitation radiation at a wavelength of 633 nm was used, the AuAg nanorods generated, within the experimental error, SERS spectra as intense as those generated by the commonly used solid Au and Ag nanorods. This means that AuAg nanorods, which are easy to synthesize and whose synthesis is less sensitive to possible surfactant contamination, can be used in SERS measurements as plasmonic nanoresonators that are as effective as Au nanorods, which are much more difficult to synthesize.

**Figure 11:**
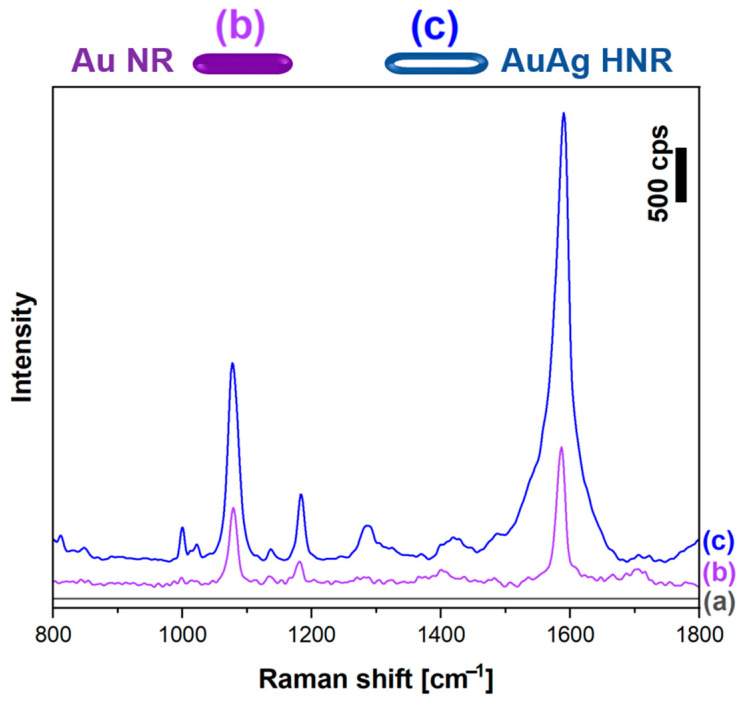
Raman spectrum of p-MBA monolayer on Pt: (a) before the deposition of plasmonic nanorods, (b) covered with solid Au nanorods and (c) covered with hollow AuAg nanorods. Excitation radiation wavelength: 532 nm.

Another significant advantage of AuAg nanorods becomes apparent when measurements are performed using excitation radiation having a much shorter wavelength. Figure 11 shows SERS spectra, which were enhanced by solid Au nanorods and hollow AuAg nanorods and were measured using excitation radiation with a wavelength of 532 nm. As can be seen in Figure 11, for this wavelength, the intensity of the SERS spectrum that was enhanced by the hollow AuAg nanorods was about 3 times larger than the intensity of the SERS spectrum that was enhanced by solid Au nanorods. This additional large SERS enhancement was probably generated by the remaining Ag nanostructure that was still present after the galvanic exchange inside the hollow AuAg nanorod—for this range of the excitation radiation, silver is a metal that generates a much higher SERS enhancement factor than gold [29,30]. This means that hollow AuAg nanorods exhibit a larger spectral range for practical applications than standard Au nanorods, making them more versatile for detecting and analyzing various molecular signatures.

### 2.5. Stability Tests of Ag and AuAg Nanorods

**Figure 12:**
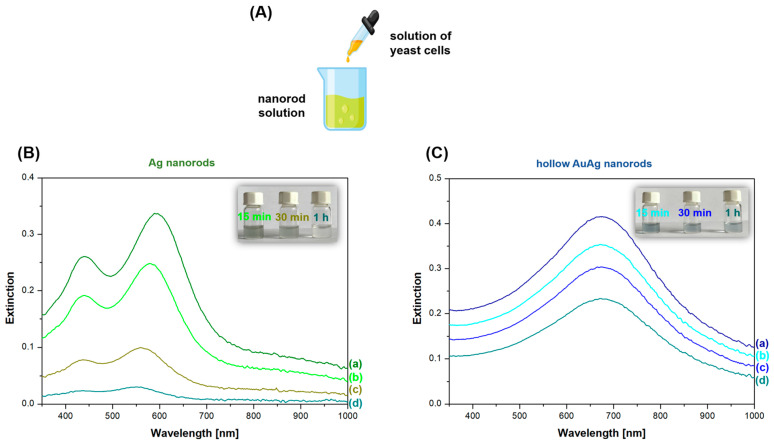
(**A**) Schematic drawing illustrating an experiment to compare the stability of Ag and AuAg nanorods. (**B**) Temporal evolution of the UV–vis spectrum of a mixture of solutions of yeast and Ag nanorods: (a) spectrum recorded immediately after mixing, (b) after 15 min, (c) after 30 min and (d) after 1 h. (**C**) Temporal evolution of the UV–vis spectrum of a mixture of solutions of yeast and AuAg hollow nanorods: (a) spectrum recorded immediately after mixing, (b) after 15 min, (c) after 30 min and (d) after 1 h. Inserts in the upper right corner in panels (**B**,**C**) show photographs of the solution of nanorods and yeast cells at various times after mixing.

A significant disadvantage of Ag nanorods, which are very active in SERS spectroscopy, is their low stability in many environments, for example, when they are in contact with certain biological samples. In order to compare the stability of solid Ag nanorods and hollow AuAg nanorods, samples of these nanoparticles were exposed to a yeast solution, and the UV–vis extinction spectra of the systems were monitored over time. The experiment started with the preparation of sols of Ag and hollow AuAg nanorods and a solution of yeast cells. In the next step, the yeast solution was added to the sols of various nanorods, and immediately after that, the UV–vis spectrum of the mixture was recorded for each case; then, the UV–vis spectra and the color of the solution were continuously observed and recorded for each mixture at 5, 15, and 30 min and 1 h after the addition of the yeast solution. As can be seen in Figure 12, the UV–vis spectrum of the solution containing AuAg nanorods was significantly more stable over time than the UV–vis spectrum of the solution containing Ag nanorods. This experiment demonstrates that AuAg nanorods exhibit significantly greater stability in the presence of a yeast solution than Ag nanorods. Their relatively high stability is probably attributable to the gold, which has high chemical stability, that formed on the outermost parts of these nanoparticles.

## 3. Materials and Methods

### 3.1. Materials

Silver nitrate, trisodium citrate dihydrate, sodium borohydride, ascorbic acid, sodium hydroxide, hexadecyltrimethylammonium bromide (CTAB), hydroxylamine hydrochloride and 4-mercaptobenzoic acid were purchased from Sigma-Aldrich (Poznań, Poland). HAuCl_4_ (a 30% solution in dilute HCl, 99.99% trace metals basis) was acquired from the Polish State Mint (Warsaw, Poland). All of the reagents were used as received, without further purification or treatment. The water used in all the experiments was purified using a Millipore Milli-Q system (Merck Millipore, Burlington, MA, USA). 

### 3.2. Synthesis of Silver Nanorods

Silver nanorods were obtained according to a slightly modified version of the procedure described by Murphy et al. [31]. The synthesis began with the obtainment of a sol of 4 nm Ag seed nanoparticles; for this purpose, 10 mL of 0.25 mM AgNO_3_ solution and 10 mL of 0.25 mM trisodium citrate solution were mixed, and then, under vigorous stirring, 0.6 mL of a 10 mM NaBH_4_ solution was quickly added. The sol of Ag seeds that was obtained was stored in a refrigerator at 4 °C for 2 h before being used in the next step of the synthesis. During the stabilisation of the sol of Ag seeds at 4 °C, what is known as a ‘growth solution’ was prepared by mixing the following solutions: 0.25 mL of 10 mM AgNO_3_, 0.5 mL of 100 mM ascorbic acid and 10 mL of 80 mM CTAB. Then, to produce rod-shaped Ag nanoparticles of different lengths, various amounts of the Ag seed sol (0.5, 0.25, 0.125 or 0.06 mL) were added to the growth solution. Finally, 0.1 mL of the 1 M NaOH solution was added, and the mixture was gently shaken.

### 3.3. Galvanic Exchange Reaction—Synthesis of AuAg Nanorods

In order to obtain AuAg nanorods, the sol of Ag nanorods that was obtained in the previous step was diluted in a 1:5 ratio, heated under gentle stirring to 60 °C, and then 30 μL of 2 mM NH_2_OH solution and 90 μL of 0.25 mM HAuCl_4_ solution were added dropwise.

### 3.4. Synthesis of Pure Gold Nanorods

As in the case of Ag nanorods, the synthesis of Au nanorods is based on growing seeds. To prepare a sol of gold seeds, solutions of the following compounds were mixed: CTAB (0.2 M, 5 mL), HAuCl_4_ (0.5 mM, 5 mL) and NaBH_4_ (10 mM, 0.6 mL). When synthesizing the Au nanorods, the growth solution was obtained by mixing solutions of the following compounds: CTAB (0.2 M, 5 mL), HAuCl_4_ (0.5 mM, 2.5 mL or 5 mL, for forming gold nanorods with a length of 45 nm and 65 nm, respectively) and ascorbic acid (78 mM, 70 μL). In the last step of the synthesis, 15 μL of the seed sol was added to the growth solution [32].

### 3.5. Experimental Techniques

A Talos F200X (Thermo Fisher, Waltham, MA, USA) transmission electron microscope (TEM) operating at 200 kV was utilized to examine the morphology of the nanomaterials that were obtained. Additionally, the elemental composition of the nanomaterials was analyzed with a Brucker BD4 energy-dispersive X-ray spectroscopy (EDS) instrument (Billerica, MA, USA).

Extinction spectroscopic measurements in the UV–vis range were conducted with a Thermo Scientific Evolution 201 spectrophotometer (Waltham, MA, USA).

The Raman measurements were carried out using a Horiba Jobin-Yvon Labram HR800 spectrometer (Palaiseau, France) equipped with a Peltier cooled charge-coupled device detector (1024 × 256 pixels), a 600 groove/mm holographic grating and an Olympus BX40 microscope (Tokio, Japan) with a long-distance 50× objective. Excitation radiation was provided at a wavelength of 532 nm using a frequency-doubled Nd:YAG laser and at a wavelength of 633 nm using a He–Ne laser.

## 4. Conclusions

Hollow gold–silver nanorods were synthesized. The EDS elemental mapping of these nanostructures showed that the distribution of gold and silver is not uniform: the gold is mainly located in the outermost parts of the nanostructures, whereas the silver mainly forms the wall of the internal cavity. The hollow gold–silver nanorods that were obtained have some unique properties that make them particularly valuable for certain scientific and technological purposes. First of all, the rod-like nanostructures generate only one clearly visible resonance plasmonic band: a longitudinal one. Hollow AuAg nanorods are highly active during SERS measurements—in experiments with red excitation radiation, the SERS enhancement factors were practically the same as those generated by solid nanorods. Moreover, with green excitation radiation, the hollow AuAg nanorods generated significantly larger SERS enhancement factors than the solid Au nanorods, an effect that is probably due to the presence of silver inside the nanostructure. The cavity in the center of the nanostructure reduces the consumption of expensive gold. Additionally, the outermost gold layer ensures that these systems are relatively stable, which is important for the durability of the nanorods in various environments. The outermost gold layer of the nanostructures that were formed should also be beneficial in enhancing biocompatibility.

In summary, although different types of SERS substrates are clearly better for many applications (for example, when high repeatability of the SERS enhancement factor is required, substrates obtained by sputtering a layer of plasmonic metal onto a regularly nanostructured substrate seem to be definitely better than plasmonic nanorods), hollow AuAg nanorods represent an interesting advancement in nanomaterial science. They contain less gold, their synthesis is clearly less sensitive to possible contamination of the surfactant than the synthesis of standard Au nanorods, and in some cases, they can enhance the spectral capabilities of SERS, thereby expanding the potential applications and reliability of SERS-based technologies. Such an extension of the capabilities of SERS spectroscopy can be expected, for example, in cases in which SERS measurements are taken inside cells, plasmonic nanoparticles are introduced into the cells, and it is not possible to use macroscopic standard SERS substrates.

## 5. Patents

As a result of the work reported in this manuscript, patent application number P.449506 was prepared.

## Data Availability

The data presented in this study are available on request from A.M.
